# Effectiveness of the delivery of interventions to prevent malaria in pregnancy in Kenya

**DOI:** 10.1186/s12936-016-1261-2

**Published:** 2016-04-18

**Authors:** Stephanie Dellicour, Jenny Hill, Jane Bruce, Peter Ouma, Doris Marwanga, Peter Otieno, Meghna Desai, Mary J. Hamel, Simon Kariuki, Jayne Webster

**Affiliations:** Department of Clinical Sciences, Liverpool School of Tropical Medicine, Liverpool, UK; Disease Control Department, London School of Hygiene and Tropical Medicine, London, UK; Malaria Branch, Kenya Medical Research Institute Centre for Global Health Research, Kisumu, Kenya; Malaria Branch, Centers for Disease Control and Prevention, Atlanta, GA USA

**Keywords:** Malaria in pregnancy, Antenatal care, Intermittent preventive treatment, Insecticide-treated nets, Systems effectiveness, Service delivery, Predictors, Kenya, Sub-Saharan Africa

## Abstract

**Background:**

Coverage with malaria in pregnancy interventions remains unacceptably low. Implementation research is needed to identify and quantify the bottlenecks for the delivery and use of these life-saving interventions through antenatal clinics (ANC).

**Methods:**

A cross-sectional study was carried out in ANC across nine health facilities in western Kenya. Data were collected for an individual ANC visit through structured observations and exit interviews with the same ANC clients. The cumulative and intermediate systems effectiveness for the delivery of intermittent preventive treatment (IPTp) and insecticide-treated nets (ITNs) to eligible pregnant women on this one specific visit to ANC were estimated.

**Results:**

Overall the ANC systems effectiveness for delivering malaria in pregnancy interventions was suboptimal. Only 40 and 53 % of eligible women received IPTp by directly observed therapy as per policy in hospitals and health centres/dispensaries respectively. The overall systems effectiveness for the receipt of IPTp disregarding directly observed therapy was 62 and 72 % for hospitals and lower level health facilities, respectively. The overall systems effectiveness for ITNs for first ANC visit was 63 and 67 % for hospitals and lower level facilities, respectively.

**Conclusion:**

This study found that delivery of IPTp and ITNs through ANC was ineffective and more so for higher-level facilities. This illustrates missed opportunities and provider level bottlenecks to the scale up and use of interventions to control malaria in pregnancy delivered through ANC. The high level of clustering within health facilities suggest that future studies should assess the feasibility of implementing interventions to improve systems effectiveness tailored to the health facility level.

**Electronic supplementary material:**

The online version of this article (doi:10.1186/s12936-016-1261-2) contains supplementary material, which is available to authorized users.

## Background

Each year 30 million pregnancies occur in areas of stable malaria transmission in sub-Saharan Africa [1]. Malaria in pregnancy can adversely affect the health of the mother and that of the unborn child and, if untreated, can lead to severe anaemia, maternal death, pregnancy loss or intrauterine growth restriction and/or preterm delivery leading to low birth weight [2]. In sub-Saharan Africa, it is estimated that about 40 % of all pregnancies would experience *Plasmodium falciparum* placental infection without malaria in pregnancy (MiP) preventive interventions, which would result in an estimated 900,000 (Credibility Interval: 530,000–1,240,000) low birth weight deliveries every year [3]. However, MiP and its adverse effects are preventable. Through the use of the lives saved tool (LiST model) developed by the global Child Health Epidemiology Reference Group [4], it has been estimated that 94,000 neonatal deaths were prevented between 2009 and 2012 through MiP interventions, and a further 200,000 could have been averted if coverage with MiP interventions were to have met the 2010 international target of 80 % [5].

WHO recommends effective case management, prevention through insecticide-treated bed-nets (ITN) and intermittent preventive treatment (IPTp) with sulfadoxine–pyrimethamine for the control of malaria in pregnancy [6], which are proven to be efficacious and cost-effective interventions delivered through the antenatal care (ANC) platform. WHO recommendations, and Kenyan national guidelines state that IPTp should be administered at every ANC visit as early as possible during the 2nd trimester. However, there is wide discrepancy between ANC coverage and MiP intervention coverage pointing to substantial missed opportunities. The Kenya Malaria Indicators Survey carried out in 2010, found that 86 % of pregnant women in Kenya received antenatal care from a medical professional [7, 8]. However, coverage of at least one dose of IPTp with SP was 46 % and only 25 % of women received two or more doses (with at least one dose received through ANC). Although the proportion of women who receive two doses of IPT during pregnancy increased from 13 % in 2007–2009 to 21 % in 2010. Only half (49 %) of pregnant women reported that they slept under an ITN the previous night in 2008–09 [7]. A recent systematic review of 98 studies undertaken in sub-Saharan Africa points to obstacles at all levels of implementation: from healthcare providers, health facility, as well as at higher levels of the health system [9]. Implementation research is needed to identify barriers to the scale-up of MiP interventions that can be used by local decision makers. The aim of this study was to measure the effectiveness of ANC to deliver IPTp and ITNs, and to gain an understanding of where bottlenecks occur in the delivery process. The study does not intend to measure coverage of MiP interventions, which would require assessment of the whole pregnancy period (i.e. after birth) at the population level.

## Methods

### Study site

The study was undertaken in the former Nyando District, Nyanza Province, in western Kenya between February and May 2010. This area encompass the Nyando, Upper and Lower Nyakach, Miwani and Muhoroni Divisions and has been fully incorporated into Kisumu County. The former Nyando District had a population of 355,800 (1999 census), with the majority of the population from the Luo ethnic group. Malaria is perennial and holo-endemic with a parasite prevalence of 18 % among pregnant women presenting for their first ANC visit [10]. HIV prevalence among women in Nyanza Province is the highest across all provinces in the country (14 %, DHS 2008–9 [7]). The former Nyando District has a total of 40 health facilities of which 24 are owned by government, five by missions, seven privately owned and four are community run.

### Study design and data collection

A cross-sectional study was conducted in nine out of ten selected health facilities using structured observation, exit interviews of an individual ANC visit with ANC clients and a health facility audit. This study was part of a larger programme under the Malaria in Pregnancy Consortium using multi-disciplinary approaches to assess bottlenecks in the scale-up of malaria in pregnancy interventions. As part of this programme, healthcare provider’s perspective was examined through in-depth interviews following completion of observations in 2011, which will be presented separately, and the community perspective through both focus group discussions [11] and a household survey [12].

### Sampling procedure

A list of all health facilities in the selected clusters for the associated household survey was compiled consisting of the health facilities that were closest to each cluster [12]. A dual-frame sampling scheme was used to purposively select the district hospital and a representative sample of nine health facilities using probability proportional to antenatal attendance. Note that community units (level 1) were not included in the sampling frame as they did not provide antenatal care or deliver IPTp nor ITNs at the time of the study. All women attending ANC at the selected health facilities were invited to take part in the study and observations and exit interviews were carried out with all consented participants, except where there were 15 or more ANC attendees on the day at the time of registration, in which case exit interviews were conducted only on every second woman who was observed which followed the registration order.

### Data collection procedure

Data on characteristics of the health facility, demographic characteristics of the staff involved in delivery of ANC services, supplies and equipment necessary for ANC available on the day of the survey as well as stock records for the previous 1 year were collected from the health facility staff person in charge (or their representative on the day of the survey) using a structured questionnaire. A trained fieldworker was stationed at different points in the health facility to follow all the ANC processes: at registration desk, in the ANC consultation room, and at the exit to follow participants to the laboratory or pharmacy and to conduct exit interviews. All ANC attendees on each day of the survey were invited to take part and, following provision of informed consent, participant characteristics were collected and all ANC processes observed and recorded using a structured checklist. The exit interviews were conducted immediately following the observations. The data collected through exit interviews included women’s knowledge of the treatments they received during ANC and on the dosing regimen of anti-malarials as well as measures of cost of prevention of malaria in pregnancy to their household.

### Data analysis

**Fig. 1:**
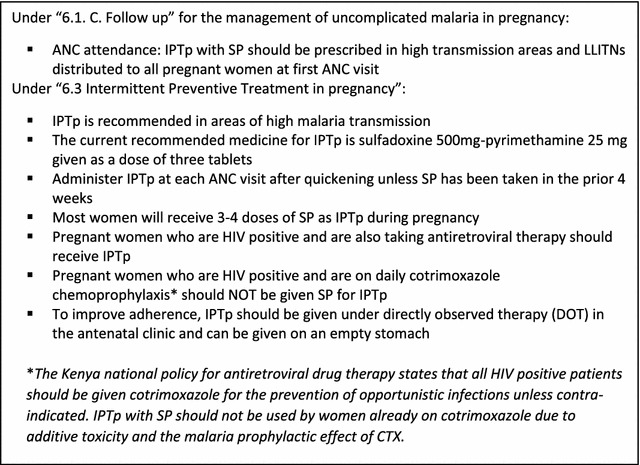
Kenya guidelines for prevention of malaria in pregnancy (2008 edition) [13]

**Fig. 2:**
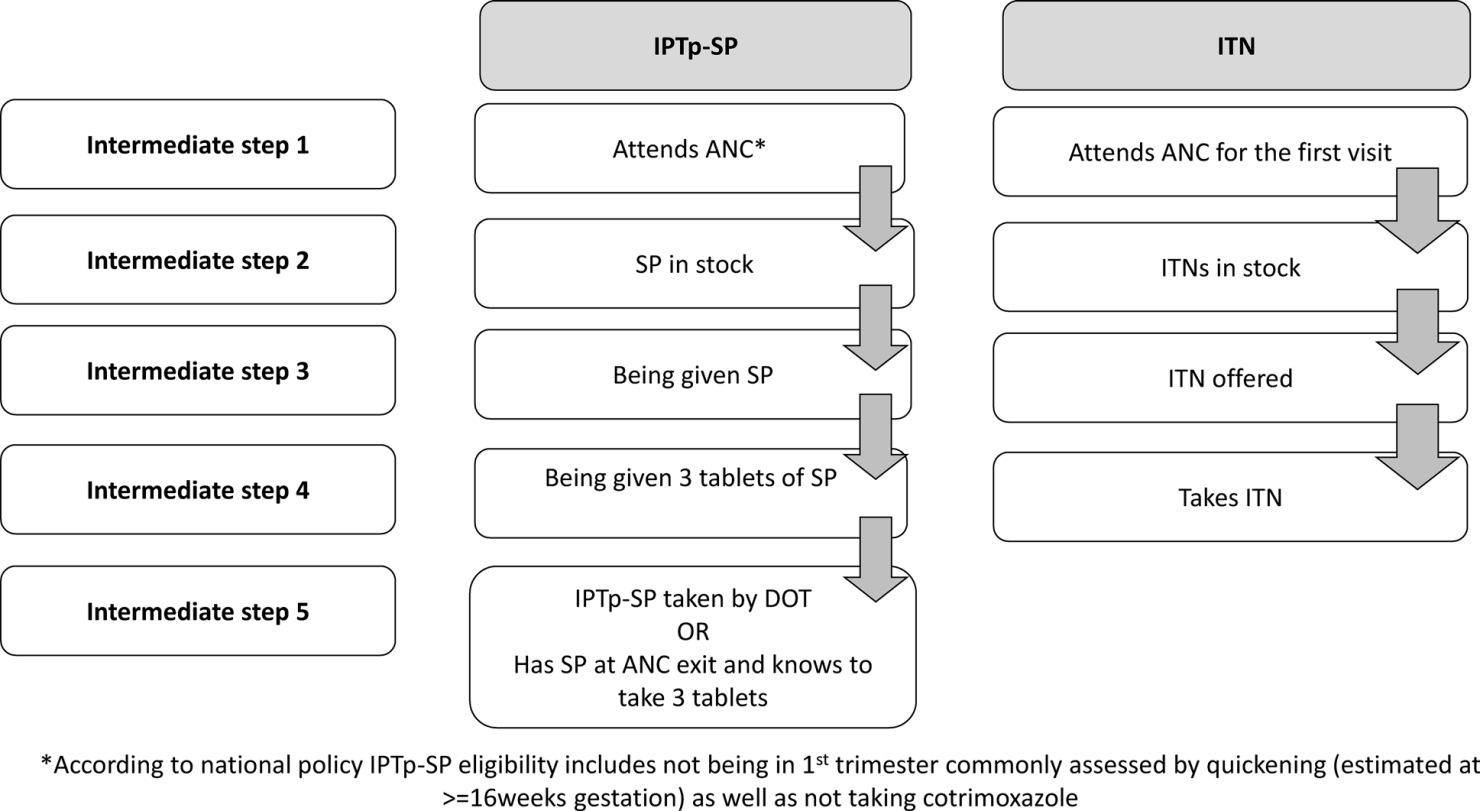
Intermediate steps in the delivery of IPTp-SP and ITN through antenatal care

Data were collected on paper forms and were double entered into EpiData (Version 3.1, EpiData Association, Odense, Denmark) and validated before being transferred to Stata (Version 13, 2013; College Station, TX) for analysis. Each intermediate step required for the effective delivery of IPTp and ITNs through ANC as per WHO guidelines [6] and Kenyan national policy [13] (Fig. 1) defined the framework for analysis (Fig. 2). The data collected were used to quantify: (1) the proportion of ANC attendees who successfully completed a designated intermediate step in the delivery of each intervention from those who completed the previous step, (2) the cumulative systems effectiveness corresponding to the proportion of eligible ANC attendees who successfully completed all intermediate steps to the designated point in the overall delivery process, and (3) design effect (DE) and intra-cluster correlation coefficients (ICC) between health facilities for each intermediate step in the delivery of IPTp and ITNs. Predictors of ineffective intermediate steps will be described in a separate paper together with results from the qualitative data.

For the IPTp effectiveness analysis, IPTp eligibility was defined as per national policy at the time of the survey to include: reporting having felt the baby move (i.e. quickening) or estimated gestational age of 16 weeks or more (to ensure the mother was well past the first trimester, when SP is contraindicated); and not being HIV positive (as a proxy for no concurrent cotrimoxazole use). As information on dates of previous ANC visits was not collected, it was not possible to assess if the last SP dose was within 1 month, but this is unlikely to be a frequent occurrence.

For ITNs, the effectiveness analyses were restricted to participants presenting for their first ANC visit for the current pregnancy. Sample weights for observations were estimated based upon the sampling probability of each selected health facility [14]. Analyses were stratified for level 4 facilities (primary hospitals) and level 2 or 3 (health centres and dispensaries) as a previous study in Mali showed that the systems effectiveness for the delivery of IPTp differed at different levels of the health system [15]. As previously defined, intermediate steps were classified as ‘ineffective’ if fewer than 80 % of participants successfully completed that step [16]. This is based on the fact that the Roll Back Malaria Partnership target for 2015 was 100 % coverage for MiP interventions, which would mean that 100 % of women should pass each step of the delivery process for all to get the interventions. A realistic goal is that at least 80 % of women receive the interventions (the target for 2010) for which each step in the delivery should be at least 80 %.

#### Intermediate steps effectiveness

The effectiveness of each intermediate step in the delivery of MiP interventions was calculated by estimating the proportion of women who successfully completed each step [15, 16]. The intermediate steps for IPTp according to policy for eligible women (not on cotrimoxazole prophylaxis for the prevention of opportunistic infections associated with HIV infection, and not administered another anti-malarial for treatment of malaria) were: (1): attend ANC consultation after quickening (estimated at around 16 weeks gestation); (2): attend ANC when and where SP is in stock; (3): be given any SP; (4): be given three tablets of SP; (5): be given SP by DOT or leaving the facility with three tablets of SP and able to report correctly how it should be taken. For ITNs the analysis was restricted to first ANC visits and there were four intermediate steps: (1) attend ANC for the first visit; (2) attend ANC when and where ITNs are in stock; (3) be offered an ITN and (4) take the ITN. Where ITN stock information was unavailable and no clients received ITNs from that facility on the day of the survey, these were classified as stock-outs for the effectiveness analysis.

The data were collected via structured observations except for ‘having three tablets of SP at exit with knowledge of correct regimen’, which was obtained through exit interviews with ANC clients.

#### Cumulative systems effectiveness

The overall cumulative systems effectiveness was estimated as the proportion of eligible pregnant women who successfully received each MiP intervention according to policy on the specific ANC visit. For IPTp two cumulative systems effectiveness estimates were calculated (1) a ‘per policy’ estimate of being given three tablets of SP by directly observed therapy (DOT) and (2) being given three tablets of SP by DOT or without DOT, but women without DOT had three tablets of SP upon leaving the health facility and knew the correct dosing to take home (and, therefore, had the potential to receive a dose of IPTp-SP).

#### The design effect and intra-cluster correlation coefficients

Design effect (DE) and intra-cluster correlation coefficients (ICC) were used to measure the level of clustering and correlation within health facilities. DE was calculated as the difference between the variance based on the clusters used compared with a modelled variance if a simple random sample was used. DEs were calculated for each intermediate step (described above and in Fig. 2) aggregated for all health facilities combined and for health facilities clustered according to level 4 or level 2 and 3 combined. ICC and DE are closely related and can be derived from one to the other using the following formula: ${\text{DE}} = {\text{ICC }} \times \left( {{\text{m}} - 1} \right) + 1$ where m is average number per cluster. This allows different ways of looking at clustering [17].

## Ethics

The study was approved by the ethics committees of the Kenya Medical Research Institute (KEMRI), the U.S. Centers for Disease Control and Prevention (CDC), the London School of Hygiene and Tropical Medicine, and the Liverpool School of Tropical Medicine. Health workers gave signed informed consent at the initial meeting for structured questionnaires and for structured observations. Pregnant women gave signed informed consent for observations and exit interviews immediately prior to beginning the ANC process. Permission from the District Health Management Teams was sought and heads of health facilities were informed before initiation of data collection.

## Results

Data collection took place between first of March and 28th of May 2010 in nine of the ten selected health facilities. The Nursing Officer in charge of one health facility refused permission to collect data. A total of 792 pregnant women were enrolled and observations were carried out for 753 participants while exit interviews were conducted with 613. For 39 participants enrolled, no observation was available as they did not receive antenatal care on the day of the survey and were therefore not included in the analysis: 23 women were sent home by the health facility staff due to lack of time either because they arrived late or because of long waiting time; six women came to the clinic but there were no ANC healthcare providers at the health facility on that day; four women were sent home as they came before their recommended ANC return visit date; two were sent to the labour ward; one was sent home as she didn’t have money to pay for the ANC laboratory tests and one was referred to another health facility with a laboratory to do the required ANC laboratory tests. Furthermore, two observations could not be carried out due to refusal from one healthcare provider and withdrawal from one participant after providing informed consent. Thirty-three participants completed part of the ANC process and were told to come back for the remaining processes on another day. These 33 women were included in the analysis. One health facility, the mission hospital, was excluded from the systems effectiveness analysis as the IPTp policy was not implemented in this facility.

### Description of health facility, healthcare providers and ANC attendees

All health facilities were government run with the exception of one health facility, which was a mission hospital but was then excluded from the analysis as described above (Table 1). There were four hospitals (level 4 facility), three health centres (level 3) and two dispensaries (level 2). The mean number of staff who usually provide ANC services was 5 (range 2–12). All facilities offered prevention of mother to child transmission of HIV (PMTCT) five days a week and the majority offered ANC 5 days a week but two facilities had a specific day in the week for ANC and did not offer ANC services on other days. Three health facilities reported having support for ANC from a non-governmental organization (NGO) or research institute and seven had support for PMTCT from NGOs. Six of the nine facilities had a laboratory, five of which had a functioning microscope and all except one facility reported having drinking water available. All had at least two supervisory visits in the last 6 months, which included a visit to ANC for all but one facility. The majority of health facilities reported getting their drugs directly from the Kenya Medical Supplies Agency (KEMSA) and all but one facility had at least one episode of SP stock-out in the last 6 months. All health facilities reported that ITNs were Olyset^®^ brand and were supplied by Population Services International (PSI), and that ITNs were offered free of charge.

**Table 1:** Description of the health facilities characteristics

Type	1	2	3	4	5	6	7	8	9
	Sub-district hospital	Sub-district hospital	Dispensary	District hospital	Mission hospital	Health centre	Dispensary	Health centre	Health centre
Level	4	4	2	4	4	3	2	3	3
Catchment population	79,200	88,000	7500	23,400	6825	13,200	8100	5808	19,800
Number of ANC visits in 2009	2734.00	1509.00	363.00	1156.00	500.00	449.00	807.00	1354.00	1038.00
Staffing									
Medical officer	1	1		1					
Clinical officer	9	2	1	6	1	1		2	1
Pharmacist	1	1		1					
Public health/registered nurse	8	8	3	12	6	6		2	3
Enrolled nurse	10	6		6	2	1	2	2	4
Assistant nurse					5				
Lab technician	3	3		2		4	2	2	
Number of staff usually working in ANC	4	2	4	4	9	5	3	4	12
Time to closest referral centre (min)	45	60	45	30	30	90	60	60	30
Laboratory	Yes	Yes		Yes	Yes			Yes	Yes
Pharmacy	Yes	Yes	Yes	Yes	Yes		Yes	Yes	Yes
Number of observation	222	115	50	99	37	42	64	92	64
Number of exit interviews	128	115	50	57	37	35	56	86	49

Overall 78 health workers were enrolled across nine health facilities (Table 2). The highest proportions (27 %) were registered nurse or midwifes, followed (14 %) medical or clinical officers; the rest included enrolled nurses, nurse students, laboratory technicians, pharmacists, nurse aids and community health workers based in the facilities. Over half (61.5 %) were female staff and the median age was 30 years (range 21–61). Of staff usually providing ANC care, 12.8 % had focused antenatal care training in last five years, 16.7 % had MiP training and 52.6 % PMTCT specific training in the same period. The mean number of years they worked in the health facility was 2.3 (median 1 year, range 0–20). The majority of staff were of Luo ethnicity (72.0 %), 10.7 % were Kalenjin, 9.3 % were Luyha and 6.7 % were Kisii.

**Table 2:** Characteristics of health workers enrolled in health facilities where ANC observations took place (number (%) unless otherwise specified)

Health facility	1	2	3	4	5	6	7	8	9	Overall
Number of staff interviewed	16	8	3	16	7	5	2	9	12	78
Number female	9 (56.3)	3 (37.5)	3 (100.0)	11 (68.8)	4 (57.1)	4 (80.0)	2 (100.0)	3 (33.3)	9 (75.0)	48 (61.5)
Mean age in years (SD)	29 (8)	34 (8)	34 (8)	31 (7)	34 (11)	34 (11)	38 (5)	37 (11)	34 (10)	33 (9)
Number resident in the district	3 (18.8)	0 (0.0)	1 (33.3)	4 (25.0)	1 (14.3)	1 (20.0)	0 (0.0)	6 (66.7)	3 (25.0)	21 (25.6)
Number from district	4 (25.0)	1 (12.5)	1 (33.3)	1 (43.8)	3 (42.9)	1 (20.0)	1 (50.0)	6 (66.7)	4 (33.3)	31 (37.8)
Ethnicity										
Luo	6 (46.2)	5 (62.5)	3 (100.0)	14 (93.3)	6 (85.7)	2 (40.0)	2 (100.0)	8 (88.9)	7 (58.3)	53 (71.6)
Luyha	4 (30.8)	0	0	0	0	0	0	1 (11.1)	2 (16.7)	7 (9.5)
Kalenjin	1 (7.7)	2 (25.0)	0	1 (6.7)	0	1 (20.0)	0	0	3 (25.0)	8 (10.8)
Kisi	2 (15.4)	1 (12.5)	0	0	1 (14.3)	1 (20.0)	0	0	0	5 (6.8)
Other	0	0	0	0	0	1 (20.0)	0	0	0	1 (1.4)
Mean years in health facility (SD)	0 (1)	2 (3)	3 (5)	2 (2)	3 (3)	0 (0)	4 (2)	7 (7)	3 (3)	2 (4)
Number working in other departments	15 (93.8)	7 (87.5)	2 (66.7)	12 (80.0)	6 (85.7)	4 (80.0)	2 (100.0)	9 (100.0)	11 (91.7)	72 (88.9)
Highest qualifications										
Medical or clinical officers	3	2	0	3	1	1	0	0	1	11
Pharmacist	1	0	0	1	0	0	0	0	0	2
Registered nurse/midwife	2	2	2	4	4	3	0	0	4	21
Enrolled nurse/midwife	1	0	1	0	0	1	0	0	3	6
Nurse aid	0	0	0	0	1	0	0	0	0	1
Laboratory technician	1	1	0	2	1	0	0	2	1	8
Student	6	0	0	0	0	0	0	0	2	8
Other	1	1	1	1	0	0	0	5	0	9
Number with FANC training in last 5 years	4 (25.0)	1 (12.5)	1 (33.3)	1 (6.25)	0 (0.0)	0 (0.0)	0 (0.0)	1 (11.1)	2 (16.7)	10 (12.2)
Number with PMTCT training in last 5 years	6 (37.5)	4 (50.0)	2 (66.7)	10 (62.5)	3 (42.9)	2 (40.0)	1 (50.0)	7 (77.8)	6 (50.0)	41 (50.0)
Number with MiP in training last 5 years	3 (8.8)	3 (37.5)	1 (33.3)	2 (12.5)	1 (14.3)	0 (0.0)	0 (0.0)	2 (2.2)	1 (9.3)	15 (15.9)

*FANC* focused antenatal care, *MiP* malaria in pregnancy, *PMTCT* prevention of mother to child transmission of HIV, *SD* standard deviation

The median age of the ANC attendees was 23 years (range 11–46) and 31 % were primigravid women (Table 3). For 43.7 % of women they participated on their first visit for the current pregnancy, for 23.4 % it was their 2nd visit, and 26.3 % presented for their 3rd or more visit. Median gestational age was 30 weeks (range 10–42) and only 1 % of women were in their first trimester. Nearly all women reported the reason for their visit to ANC was to receive routine care (95.8 %) and a few reported coming for both routine ANC and because of feeling unwell (2.8 %). The majority of ANC attendees were Luo (84.0 %) followed by Kalenjin (7 %) and Luyha (4.2 %).

**Table 3:** Characteristics of pregnant women (number (%) unless otherwise specified)

	Level 4	Level 3	Level 2	Overall
Number	473	198	114	785
Mean age (SD)	23.8 (5.9)	23.5 (5.6)	24.7 (6.8)	23.9 (6.0)
Age group				
<15 years	4 (0.9)	3 (1.5)	2 (1.8)	9 (1.2)
15–20 years	117 (24.7)	48 (24.2)	28 (24.6)	193 (24.6)
20–29 years	269 (56.9)	114 (57.6)	57 (50.0)	440 (56.1)
30–39 years	75 (15.9)	30 (15.2)	22 (19.3)	127 (16.2)
40–49 years	8 (1.69)	1 (0.5)	4 (3.5)	13 (1.7)
≥50 years	0 (0.0)	2 (1.0)	1 (0.9)	3 (0.4)
Marital status				
Single	77 (16.5)	40 (20.2)	17 (15.0)	134 (17.3)
Married	384 (82.4)	154 (77.8)	92 (81.4)	630 (81.2)
Divorced	2 (0.4)	3 (1.5)	4 (3.5)	9 (1.2)
Widowed	3 (0.6)	1 (0.5)	0 (0.0)	4 (0.5)
Highest level of education				
None	144 (30.4)	84 (42.4)	70 (61.4)	298 (38.0)
Primary	271 (57.3)	99 (50.0)	38 (33.3)	408 (52.0)
Secondary	41 (8.7)	11 (5.6)	5 (4.4)	57 (7.3)
Higher education	17 (3.6)	4 (2.0)	1 (0.9)	22 (2.8)
Gravidity				
Primi	145 (30.7)	71 (35.9)	27 (23.7)	243 (31.0)
2–3	272 (57.5)	101 (51.0)	56 (49.1)	429 (54.7)
4+	56 (11.8)	26 (13.1)	31 (27.2)	113 (14.4)
Number of children under 5				
0	209 (44.6)	85 (43.2)	35 (31.5)	329 (42.3)
1	141 (30.1)	65 (33.0)	37 (33.3)	243 (31.3)
2–4	117 (25.0)	45 (22.8)	39 (35.1)	201 (25.9)
5+	2 (0.4)	2 (1.0)	0 (0.0)	4 (0.5)
Socioeconomic status group				
Poorest	44 (13.1)	28 (16.5)	51 (48.1)	123 (20.1)
Very poor	54 (16.0)	50 (29.4)	19 (17.9)	123 (20.1)
Poor	63 (18.7)	45 (26.5)	14 (13.2)	122 (19.9)
Less poor	79 (23.4)	29 (17.1)	15 (14.2)	123 (20.1)
Least poor	97 (28.8)	18 (10.6)	7 (6.6)	122 (19.9)
* Missing* ^*a*^	*136*	*28*	*8*	*172*
Gestation trimester				
First	12 (2.5)	5 (2.5)	3 (2.6)	20 (2.6)
2nd	155 (32.8)	68 (34.3)	36 (31.6)	259 (33.0)
3rd	228 (48.2)	98 (49.5)	67 (58.8)	393 (50.1)
* Missing*	*78 (16.5)*	*27 (13.6)*	*8 (7.0)*	*113 (14.4)*
ANC visit number				
1	190 (40.2)	111 (55.8)	43 (37.4)	344 (43.7)
2	117 (24.7)	35 (17.6)	32 (27.8)	184 (23.4)
3	82 (17.3)	22 (11.1)	19 (16.5)	123 (15.6)
4	32 (6.8)	8 (4.0)	12 (10.4)	52 (6.6)
5	6 (1.3)	2 (1.0)	0 (0.0)	8 (1.0)
6	3 (0.6)	2 (1.0)	0 (0.0)	5 (0.6)
* Missing*	*43 (9.1)*	*19 (9.6)*	*9 (7.8)*	*71 (9.0)*
Reason for visit				
Routine ANC	325 (95.9)	162 (95.3)	102 (96.2)	587 (95.8)
Routine ANC + ill	2 (0.6)	3 (1.8)	1 (0.9)	6 (1.0)
Ill	10 (3.0)	4 (2.4)	3 (2.8)	17 (2.8)
* Missing*	*136*	*29*	*8*	*175*

^a^Information on assets was only collected for participants completing exit interviews

*ANC* antenatal care; *SD* standard deviation

### Systems effectiveness of delivery of IPTp-SP

**Fig. 3:**
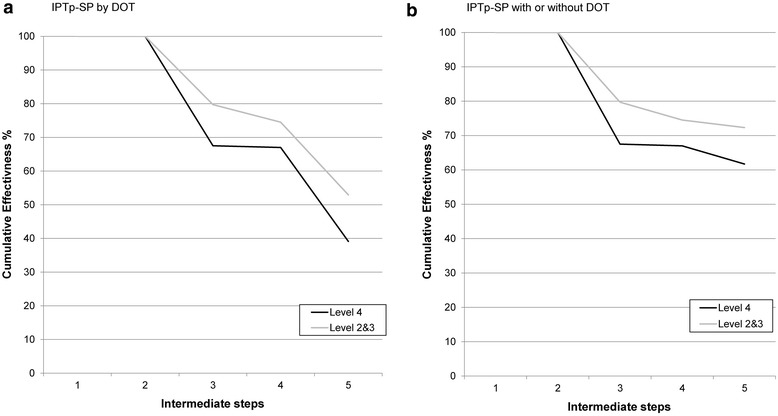
**a** Cumulative system effectiveness for the delivery of IPTp-SP by DOT through ANC and **b** Cumulative system effectiveness for the delivery of IPTp-SP through ANC either by DOT or pregnant women having three tablets of SP at exit and knowing how to take them. Intermediate steps are as follows: *step 1*, Eligible pregnant women attend ANC in her second trimester; *step 2*, SP is in stock; *step 3*, SP is given to the pregnant women; *step 4*, the correct dose of SP is given (three tablets); *step 5*, the pregnant women take IPTp-SP by DOT (**a**) or either by DOT or pregnant women having three tablets of SP at exit and knowing how to take them (**b**)

Out of the 748 observations in the eight health facilities, 546 participants were eligible for IPTp according to national policy (Table 4). Of the 202 not eligible, 117 (16 %) were HIV positive and assumed to be taking daily cotrimoxazole, 14 had a gestation <16 weeks and/or hadn’t felt the baby move, and gestational age was unknown for 86 participants (note these are not mutually exclusive categories). Of the eligible women, only 45.8 % were given IPTp by DOT per policy. This was similar for level 4 (39.8 %) and levels 2 and 3 combined (53.3 %) (Fig. 3**a**). Two intermediate steps were identified as ineffective in the delivery of IPTp in level 4 facilities, namely: being given any SP (74.0 %) and being given IPTp by DOT (67.3 %). For health facilities level 2 and 3, the only ineffective step was being given IPTp by DOT (70.1 %) as 80.3 % of eligible women were given SP during their ANC visit.

**Table 4:** Intermediate and cumulative system effectiveness for the delivery of IPTp with and without directly observed therapy (DOT) through the antenatal care platform

	Level 4			Levels 3 and 2		
	n	Intermediate	Cumulative	n	Intermediate	Cumulative
		% (95 % CI)	% (95 % CI)		% (95 % CI)	% (95 % CI)
With DOT						
IPTp eligible^a^	304			242		
SP in stock	304	100.0	100.0	242	100.0	100.0
Receive SP during visit	208	*68.6 [60.2, 75.9]*	68.6 [60.2, 75.9]	198	80.3 [61.7, 91.1]	80.3 [61.7, 91.1]
Receive three doses SP during visit	206	99.0 [96.1, 99.7]	67.8 [60.6, 74.3]	187	94.1 [77.5, 98.7]	75.5 [55.4, 88.5]
Took SP by DOT	121	*64.6 [22.2, 92.1]*	39.8 [12.5, 75.4]	129	*70.1 [22.1, 95.1]*	53.3 [21.0, 83.1]
With or without DOT^b^						
IPTp eligible^a^	208			220		
SP in stock	208	100.0	100.0	220	100.0	100.0
Being given SP during visit	140	*67.5 [60.3, 74.0]*	67.5 [60.3, 74.0]	181	79.7 [60.8, 90.9]	79.7 [60.8, 90.9]
Being given three doses SP during visit	139	99.2 [96.2, 99.9]	67.0 [60.6, 72.8]	170	93.4 [74.5, 98.6]	74.5 [53.6, 88.1]
Has SP on exit	52	88.0 [63.3, 96.9]	40.3 [18.2, 67.2]	50	93.4 [45.9, 99.6]	54.0 [17.6, 86.5]
Knows to take three tablets SP	48	92.1 [78.1, 97.5]	37.2 [17.7, 61.9]	49	96.4 [59.6, 99.8]	52.5 [16.3, 86.2]
SP by DOT	80	*58.3 [19.3, 89.1]*	39.1 [15.9, 68.6]	118	*71.0 [22.9, 95.3]*	52.9 [20.2, 83.2]
Knows to take three tablets SP or took SP as DOT	128	92.1 [87.4, 95.1]	61.7 [55.7, 67.4]	167	97.1 [70.6, 99.8]	72.3 [46.9, 88.6]

^a^IPTp eligibility according to Kenya national guidelines was women not taking cotrimoxazole (or being HIV positive as a proxy for cotrimoxazole use), having felt the baby move (i.e. past quickening) or being 16 weeks gestation or over

^b^Analysis limited to participants completing exit interviews where information on availability of SP at exit was collected

*ANC* antenatal care; *CI* confidence interval, *DOT* directly observed therapy, *IPTp* intermittent preventive treatment *SP* sulfadoxine–pyrimethamine

When either having SP at exit and having the knowledge of how to take the three tablets at home or taking IPTp by DOT were considered as the final step in the delivery process, only the 576 participants with exit interviews were included. Of these, 428 were eligible for IPTp. The cumulative effectiveness was higher at 61.7 % for level 4 and 72.3 % for levels 2 and 3 combined respectively (Fig. 3b) and the only ineffective step in the delivery process was being given any SP at level 4 facilities (Table 4).

### Systems effectiveness for delivery of ITNs

**Fig. 4:**
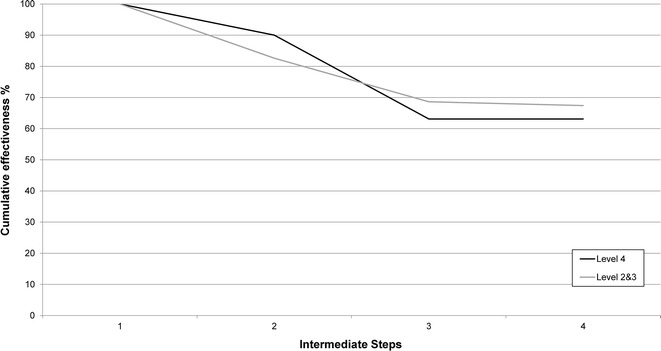
Cumulative system effectiveness for the delivery of ITN through ANC stratified for health facility level 4 and 2/3 combined. Intermediate steps are as follows: *step 1* attend ANC for first visit;* step 2* ITN are in stock;* step 3* an ITN is offered to the women;* step 4* the women accepts and takes the ITN

The overall cumulative effectiveness for the delivery of ITN during a first ANC visit was 65.3 % which is better than for the delivery of IPTp per policy (Table 5). Hospitals (level 4 health facilities) performed similarly to dispensaries and health centres combined (levels 2 and 3 respectively) where 63.1 and 67.4 % of first ANC attendees respectively were offered and took an ITN (Fig. 4). The ineffective step in the ITN delivery process for level 4 facilities was a woman being offered an ITN. Despite the cumulative effectiveness for level 2 and 3 facilities not reaching 80 %, all intermediate steps were considered effective (completed by >80 % of women). Stock-outs of ITNs was recorded in four of the eight health facilities included in this analysis restricted to first ANC visits, varying from 1 to 4 days of the survey.

**Table 5:** Intermediate and cumulative system effectiveness for the delivery of ITN at first antenatal care visit

Steps for ITN delivery	Level 4			Levels 3 and 2		
	n	Intermediate	Cumulative	n	Intermediate	Cumulative
		% (95 % CI)	% (95 % CI)		% (95 % CI)	% (95 % CI)
Attend ANC	142	100.0		135	100.0	
ITN in stock	127	90.0 [76.9, 96.1]	90.0 [76.9, 96.1]	115	82.6 [47.6, 96.1]	82.6 [47.6, 96.1]
Given ITN during consultation by healthcare provider	91	*70.1 [54.3, 82.2]*	63.1 [55.9, 69.7]	94	83.0 [61.2, 93.8]	68.6 [38.0, 88.6]
Women took ITN	91	100	63.1 [55.9, 69.7]	93	98.4 [81.8, 99.9]	67.4 [35.6, 88.6]

*ANC* antenatal care, *CI* confidence interval, *ITN*, insecticide-treated net

### Clustering and design effects for each intermediate delivery step

Table 6 shows the design effects and intra-cluster correlation for each intermediate step in the delivery of IPTp and ITNs. Overall design effects and the clustering at health facility level were higher when looking at all health facilities and for levels 2 and 3 facilities combined compared to level 4 alone. For IPTp, two intermediate steps showed a high level of clustering: being given IPTp by DOT and having three tablets of SP when leaving the facility. This clustering and high design effect was apparent across all groups (overall, level 4 and levels 2 and 3 combined, respectively). For the delivery of ITNs, the design effect for the intermediate step of being offered an ITN was high for facility levels 2 and 3 combined but not for level 4. This is further illustrated in Additional file 1: Tables S1, S2, showing some facilities performing very well and others failing to deliver the interventions adequately. In particular, one dispensary delivered IPTp by DOT to 94 % of eligible clients and 100 % of clients coming for their first visit received an ITN. At the other end of the spectrum, there were a high number of missed opportunities in one health centre with close to 70 % of eligible clients leaving the facility without receiving any SP for IPTp (despite SP being in stock), no women receiving IPTp by DOT and 65 % of first visits leaving ANC without an ITN.

**Table 6:** Design effect and intra-cluster correlation for each intermediate step for the delivery of ITN and IPTp

Delivery steps	Level 4				Level 3 and 2				Overall			
	% Pregnant women	% Range	DE	ICC	% Pregnant women	% Range	DE	ICC	% Pregnant women	% Range	DE	ICC
ITN eligible: attend ANC first visit	100.0				100				100			
ITN in stock	90.9	(84.9–100.0)	3.1	0.023	100				94.8	(84.9–100.0)	5.1	0.044
Offered an ITN	63.4	(55.6–74.2)	0.7	−0.003	68.6	(35.3–100.0)	9.1	0.087	66.0	(35.3–100.0)	4.4	0.037
IPTp eligible^a^	100.0				100				100			
SP in stock	100.0				100				100			
Seen at ANC consultation	100.0	100.0			99.7	(98.6–100.0)	0.7	−0.003	99.9	(98.5–100.0)	0.9	-0.001
Being given SP during visit	68.6	(61.5–73.2)	2.2	0.013	80.3	(60.7–95.7)	6.8	0.062	74.0	(60.7–95.7)	4.6	0.039
Being given three tabs SP during visit	99.0	(98.2–100.0)	0.9	−0.001	94.1	(76.5–100.0)	5.1	0.044	96.5	(46.4–95.7)	4.9	0.042
SP by DOT	64.6	(0.0–86.9)	38.0	0.398	70.1	(24.1–100.0)	34.2	0.357	67.3	(24.1–100.0)	29.0	0.301
Has SP on exit	36.7	(11.3–96.6)	26.0	0.269	27.1	(0–80.0)	28.7	0.298	31.5	(0–96.6)	22.3	0.229
Knows to take three tablets SP	92.1	(85.7–100.0)	1.0	0.000	96.4	(87.5–100.0	2.7	0.018	94.2	(85.7–100.0)	1.7	0.008
Knows to take three tablets SP or took SP as DOT	92.1	(86.2–93.8)	0.6	−0.004	97.1	(70.0–100.0)	6.4	0.058	94.8	(70.0–100.0)	2.9	0.020

^a^IPTp eligibility according to Kenya national guidelines was women not taking cotrimoxazole (or being HIV positive as a proxy for cotrimoxazole use), having felt the baby move (i.e. past quickening) or being 16 weeks gestation or over

*ANC* antenatal care, *DE* design effect, *DOT* directly observed therapy, *ICC* intra-class correlation, *IPTp* intermittent preventive treatment, *ITN* insecticide-treated net, *SP* sulfadoxine–pyrimethamine

## Discussion

This study found that the systems effectiveness of ANC to deliver IPTp was unacceptably low and only slightly better for ITNs. The majority of health facilities failed to deliver these interventions to 80 % of eligible women. The findings illustrate clear missed opportunities for protecting women and their unborn babies from the adverse effects of MiP and the need to maximize the public health impact of these cost-effective interventions.

Despite presenting to ANC and being eligible for IPTp, over 54 % of pregnant women did not receive a dose of IPTp by DOT and 33 % did not receive any SP (either by DOT or to take home) despite SP being in stock. The system effectiveness was slightly better for health centres and dispensaries compared to hospitals with 80 % of eligible women receiving some SP for IPTp, although only 53 % received the correct dose by DOT overall, compared to 62 and 40 % in hospitals for any IPTp and IPTp by DOT, respectively. Although stock-out of SP has been reported as a major issue in previous studies, in this study no stock-outs were observed on the days of visit despite the fact that facilities reported SP stock-outs in the preceding 12 months [9]. These cumulative system effectiveness estimates cannot be compared to overall IPTp coverage (i.e. 25 % for Kenya in 2010 [8] or 17 % for Nyanza Province [7]) as the presented estimates are derived from a cross-section of pregnant women not capturing the IPTp coverage for the whole pregnancy period and represent IPTp eligible women coming to ANC whereas estimates from demographic health surveys and/or malaria indicator surveys are based on household surveys involving women who gave birth in the previous years disregarding if they have been to ANC or if they had a contraindication, such as taking cotrimoxazole and fail to capture pregnancy not ending in a live births (estimate close to 30 % of pregnancies for Kenya [1]). The system effectiveness for delivery of ITNs was better than for IPTp, but still underlines missed opportunities with close to 35 % of first ANC attendees leaving a health facility without an ITN. Even though all the intermediate steps for the delivery of ITNs (namely presenting for a first ANC visit; ITNs being in stock at ANC on the day; being offered an ITN and taking the ITN), were effective for health centres and dispensaries, only 67 % of women coming for a first ANC visit left these facilities with an ITN. In hospitals, only 70 % of women were offered an ITN when they were in stock and the cumulative effectiveness was 63 %. Furthermore, missed opportunities were highlighted by 37 women (5 %) who presented to the clinic but didn’t receive ANC services for a variety of reasons ranging from late arrival at ANC, absent ANC healthcare providers, presenting before the recommended ANC return visit date, and need for ANC laboratory tests that were not available at the facility. Thirty-three (4 %) women didn’t complete their ANC visit, mainly due to delays at the laboratory, and were asked to come back the next working day. Of these only 3 % received IPTp and 12 % received an ITN. It is unknown what proportion of women returned to complete their visit as scheduled by the healthcare provider, but given the known barriers for attending ANC (such as commitment to farming, childcare, employment and transport cost) this is likely to be a small proportion [9].

A household cross-sectional study conducted concurrently alongside the health facility survey [12] found low IPTp coverage, with 59 % of recently pregnant women who attended ANC at least once between 4 and 9 months gestation reporting to have received one dose of SP. The household survey showed that among women who visited ANC at least twice during pregnancy 27 % received two doses of IPTp and only 14 % by DOT. Due to the cross-sectional nature of the health facility survey, the authors were unable to assess the proportion of pregnant women receiving at least two doses of IPTp during pregnancy, which requires data for the whole pregnancy period. Findings from the household survey suggest that the major bottlenecks occur at the health facility level, as reflected by the discrepancy between the proportions of women who attended ANC at least twice in an eligible gestation (78 %) and those receiving at least two doses of IPTp (27 %). Three quarter of participants who received either dose of IPTp said they received it by DOT, which is higher than the proportion observed in the health facility survey (58 % for hospitals and 65 % for health centres and dispensaries combined).

A related study evaluating the health systems effectiveness of ANC to deliver MiP interventions in Mali [15, 18] found that delivery of IPTp through ANC was ineffective, with 0 and 25 % of eligible women receiving IPTp by DOT at the district and community levels, respectively. The cumulative systems effectiveness of being given IPTp with or without DOT was also better in Mali, at 56 and 67 % for the district and community levels respectively. Despite the different study/country contexts, such as the higher HIV prevalence in Kenya and differing national guidelines on IPTp, similar cumulative effectiveness were observed in Kenya, with 62 % for hospitals and 72 % for health centres and dispensaries. As with the Kenya findings, the ineffective steps in Mali were being given any SP and receiving IPTp by DOT, with higher-level facilities having the least effective delivery.

Similar to the Mali study, there was a significant clustering of effective and ineffective steps and outcomes by health facility as reflected by the high design effects. Steps such as receiving IPTp by DOT or being given SP to take home varied across facilities with some facilities always and others never giving IPTp by DOT. This clustering calls attention to the need for targeted interventions to improve the delivery of MiP interventions at the health facility level. These could be implemented through enhanced supervisory visits to selected facilities.

This study provides insights on the health facility level barriers to effective delivery of MiP interventions, however, several limitations should be noted. First, the approach used in the study presented here differed from the health facility survey carried out in Mali in that survey staff were placed at different stations in ANC to capture all consenting women on the day of the survey. In Mali, each enrolled participant was followed by one study staff from the beginning of the ANC process until they exited the clinic. Although in Kenya all women could be observed on the day, in busy health facilities not all participants could be interviewed at exit as the turnover was usually faster than the time it took to complete the interview.

Secondly, the authors were not able to exclude women who received SP within the last 4 weeks which could under-estimate the cumulative systems effectiveness by including IPTp ineligible participants in the analysis. Assuming about a third of pregnant women make the minimum recommended number of four visits (34 % according to the concurrent household survey [12]), this could have been the case for some of the revisits. Also participants with unknown gestational age (4 %) could not be included in the IPTp effectiveness analysis as their eligibility could not be established. However, including these participants assuming that they were beyond the first trimester (median gestation at first ANC visit was 5 months in household survey, well into the second trimester [12]) only slightly affect the cumulative effectiveness of receiving IPTp by DOT (it was 42 % for hospitals and 52 % for lower level facilities compared to 40 and 55 % overall, respectively, and the cumulative effectiveness for with or without DOT were 55 and 74 % compared to 62 and 72 %, respectively).

ITN stock information was not available for nine survey days from two health facilities for which stock-out was assumed, however if ITNs were in stock on those days the cumulative effectiveness would not have been affected but the intermediate step of being offered an ITN in health centres and dispensaries becomes ineffective at 69 %. There could have been errors in data collection as data from observations may reflect the Hawthorne effect [19], data from exit interviews from social desirability bias [20], and data extracted from ANC registers may have been incomplete or erroneously abstracted. However, given the low observed cumulative effectiveness for each of the endpoints it seems unlikely that these factors would have an impact on the outcomes and, if anything, would over-estimate ANC delivery effectiveness. Lastly, the selected mission hospitals could not be included as one facility did not implement the policy for IPTp, and ITNs and SP for IPTp were out of stock for the whole study period and the other mission hospital refused to take part in the study. There is limited information on quality of care or system effectiveness of ANC for delivery of MiP interventions in these settings. It will be important for future studies to be inclusive of the mission run health facilities and private sector, given that care in that sector is growing and it provides health services to a significant proportion of the population in sub-Saharan Africa [21].

## Conclusion

This study identified that the delivery of MiP interventions through the ANC platform in this setting—namely the delivery of IPTp and ITNs in hospitals, and of IPTp by DOT at all health facility levels—was ineffective. The substantial variation observed between health facilities suggests that future intervention studies to increase uptake of IPTp and ITNs, as well as other ANC services, should focus on identifying facility level problems and evaluating customized solutions. Special attention should be given to the private sector, for which very little information on ANC and quality of care in general is available. Overall, the ANC systems effectiveness for delivering MiP interventions was suboptimal and highlights missed opportunities to protect pregnant women and their babies from the adverse consequences of malaria. These are cost-effective interventions with the potential of saving many lives. Qualitative data collected alongside the quantitative data in this survey will provide further insights on the reasons for these missed opportunities and potential tailored interventions.

## Additional file

10.1186/s12936-016-1261-2 Health facility performance for the delivery of IPTp with and without directly observed therapy (DOT) through the antenatal care platform. **Table S2.** Health facility performance for the delivery of ITN through the antenatal care platform at the time of the first visit.

## Abbreviations

ANC: antenatal clinic
DOT: directly observed therapy
IPTp: intermittent preventive treatment in pregnancy
ITN: insecticide-treated net
LMP: last menstrual period
MiP: malaria in pregnancy
PCA: principal component analysis
PMTCT: prevention of mother to child transmission of HIV
SES: socio-economic status
SP: sulphadoxine-pyrimethamine
WHO: World Health Organization
