# Supplementary material for: Effectiveness of the delivery of interventions to prevent malaria in pregnancy in Kenya
Source: Malar J. 2016 Apr 18;15:221. doi: 10.1186/s12936-016-1261-2 (PMC4835845; doi:10.1186/s12936-016-1261-2)
Supplement: Supplementary file 1 — 10.1186/s12936-016-1261-2 Health facility performance for the delivery of IPTp with and without directly observed therapy (DOT) through the antenatal care platform. Table S2. Health facility performance for the delivery of ITN through the antenatal care platform at the time of the first visit. [file 12936_2016_1261_MOESM1_ESM.docx]

# Supplemental Tables

**Table S1. Health facility performance for the delivery of IPTp with and without directly observed therapy (DOT) through the antenatal care platform.**

| **Health Facility** | **1** | | | **2** | | | **3** | | | **4** | | | **6** | | | **7** | | | **8** | | | **9** | | |
| --- | --- | --- | --- | --- | --- | --- | --- | --- | --- | --- | --- | --- | --- | --- | --- | --- | --- | --- | --- | --- | --- | --- | --- | --- |
|  |  | **Inter.** | **Cum.** |  | **Inter.** | **Cum.** |  | **Inter.** | **Cum.** |  | **Inter.** | **Cum.** |  | **Inter.** | **Cum.** |  | **Inter.** | **Cum.** |  | **Inter.** | **Cum.** |  | **Inter.** | **Cum.** |
| **IPTp with DOT** | **n** | **%** | **%** | **n** | **%** | **%** | **n** | **%** | **%** | **n** | **%** | **%** | **n** | **%** | **%** | **n** | **%** | **%** | **n** | **%** | **%** | **n** | **%** | **%** |
| IPTp eligible* | 149 |  |  | 78 |  |  | 47 |  |  | 77 |  |  | 28 |  |  | 53 |  |  | 69 |  |  | 45 |  |  |
| SP in stock | 149 | 100.0 | 100.0 | 78 | 100.0 | 100.0 | 47 | 100.0 | 100.0 | 77 | 100.0 | 100.0 | 28 | 100.0 | 100.0 | 53 | 100.0 | 100.0 | 69 | 100.0 | 100.0 | 45 | 100.0 | 100.0 |
| Receive SP during visit | 109 | 73.2 | 73.2 | 48 | 61.5 | 61.5 | 45 | 95.7 | 95.7 | 51 | 66.2 | 66.2 | 17 | 60.7 | 60.7 | 40 | 75.5 | 75.5 | 65 | 94.2 | 94.2 | 31 | 68.9 | 68.9 |
| Receive 3 doses SP during visit | 107 | 98.2 | 71.8 | 48 | 100.0 | 61.5 | 45 | 100.0 | 95.7 | 51 | 100.0 | 66.2 | 13 | 76.5 | 46.4 | 40 | 100.0 | 75.5 | 58 | 89.2 | 84.1 | 31 | 100.0 | 68.9 |
| Took SP by DOT | 93 | 86.9 | 62.4 | 28 | 58.3 | 35.9 | 44 | 97.8 | 93.6 | 0 | 0.0 | 0.0 | 0 | 0.0 | 0.0 | 40 | 100.0 | 75.5 | 14 | 24.1 | 20.3 | 31 | 100.0 | 68.9 |
| **IPTp with or without DOT**** |  |  |  |  |  |  |  |  |  |  |  |  |  |  |  |  |  |  |  |  |  |  |  |  |
| IPTp eligible* | 88 |  |  | 78 |  |  | 47 |  |  | 42 |  |  | 23 |  |  | 47 |  |  | 66 |  |  | 37 |  |  |
| SP in stock | 88 | 100.0 | 100.0 | 78 | 100.0 | 100.0 | 47 | 100.0 | 100.0 | 42 | 100.0 | 100.0 | 23 | 100.0 | 100.0 | 47 | 100.0 | 100.0 | 66 | 100.0 | 100.0 | 37 | 100.0 | 100.0 |
| Being given SP during visit | 63 | 71.6 | 71.6 | 48 | 61.5 | 61.5 | 45 | 95.7 | 95.7 | 29 | 69.1 | 69.1 | 14 | 60.8 | 60.8 | 36 | 76.6 | 76.6 | 62 | 93.9 | 93.9 | 24 | 66.7 | 66.7 |
| Being given 3 doses SP during visit | 62 | 98.4 | 70.5 | 48 | 100.0 | 61.5 | 45 | 100.0 | 95.7 | 29 | 100.0 | 69.1 | 14 | 100.0 | 60.8 | 36 | 100.0 | 76.6 | 55 | 88.7 | 83.3 | 24 | 100.0 | 66.7 |
| SP by DOT | 52 | 83.9 | 59.1 | 28 | 58.3 | 35.9 | 44 | 97.8 | 93.6 | 0 | 0.0 | 0.0 | 0 | 0.0 | 0.0 | 36 | 100.0 | 76.6 | 14 | 25.5 | 21.2 | 24 | 100.0 | 66.7 |
| Has SP on exit | 7 | 70.0 | 19.4 | 17 | 85.0 | 34.0 | 1 | 100.0 | 33.3 | 28 | 96.6 | 66.7 | 8 | 80.0 | 52.1 | 0 | 0.0 | 0.0 | 41 | 100.0 | 78.8 | 0 | 0.0 | 0.0 |
| Knows to take 3 tablets SP | 6 | 85.7 | 16.7 | 17 | 100.0 | 34.0 | 1 | 100.0 | 33.3 | 25 | 89.3 | 59.5 | 7 | 87.5 | 30.4 | 0 | 0.0 | 0.0 | 41 | 100.0 | 78.8 | 0 | 0.0 | 0.0 |
| Knows to take 3 tablets SP or took SP as DOT | 58 | 93.6 | 65.9 | 45 | 93.8 | 57.7 | 45 | 100.0 | 95.7 | 25 | 86.2 | 59.5 | 7 | 70.0 | 30.4 | 36 | 100.0 | 76.6 | 55 | 100.0 | 83.3 | 24 | 100.0 | 64.9 |
| \| * IPTp eligibility according to Kenya national guidelines was women not taking cotrimoxazole (or being HIV positive as a proxy for cotrimoxazole use), having felt the baby move (i.e. past quickening) or being 16 weeks gestation or over. \|  \| \| --- \| --- \| \| ** Analysis limited to participants completing exit interviews where information on availability of SP at exit was collected \|  \| \| Abbreviations: ANC, antenatal care; Cum, cumulative; DOT, directly observed therapy; Inter, intermediate; IPTp, intermittent preventive treatment; SP, sulfadoxine-pyrimethamine \|  \| | | | | | | | | | | | | | | | | | | | | | | | | |

**Table S2. Health facility performance for the delivery of ITN through the antenatal care platform at the time of the first visit.**

| **Health Facility** | **1** | | | **2** | | | **3** | | | **4** | | | **6** | | | **7** | | | **8** | | | **9** | | |
| --- | --- | --- | --- | --- | --- | --- | --- | --- | --- | --- | --- | --- | --- | --- | --- | --- | --- | --- | --- | --- | --- | --- | --- | --- |
|  |  | **Inter.** | **Cum.** |  | **Inter.** | **Cum.** |  | **Inter.** | **Cum.** |  | **Inter.** | **Cum.** |  | **Inter.** | **Cum.** |  | **Inter.** | **Cum.** |  | **Inter.** | **Cum.** |  | **Inter.** | **Cum.** |
|  | **n** | **%** | **%** | **n** | **%** | **%** | **n** | **%** | **%** | **n** | **%** | **%** | **n** | **%** | **%** | **n** | **%** | **%** | **n** | **%** | **%** | **n** | **%** | **%** |
| Attend ANC | 75 |  |  | 36 |  |  | 23 |  |  | 31 |  |  | 17 |  |  | 17 |  |  | 41 |  |  | 37 |  |  |
| ITN in stock | 65 | 86.7 | 86.7 | 36 | 100.0 | 100.0 | 23 | 100.0 | 100.0 | 26 | 83.9 | 83.9 | 8 | 47.1 | 47.1 | 17 | 100.0 | 100.0 | 41 | 100.0 | 100.0 | 26 | 70.3 | 70.3 |
| Given ITN during consultation by healthcare provider | 48 | 73.9 | 64.0 | 20 | 55.6 | 55.6 | 23 | 100.0 | 100.0 | 23 | 88.5 | 74.2 | 6 | 75.0 | 35.3 | 17 | 100.0 | 100.0 | 30 | 73.2 | 73.2 | 18 | 69.2 | 48.6 |
| Women took ITN | 48 | 100.0 | 64.0 | 20 | 100.0 | 55.6 | 23 | 100.0 | 100.0 | 23 | 100.0 | 74.2 | 5 | 83.3 | 35.3 | 17 | 100.0 | 100.0 | 30 | 100.0 | 73.2 | 18 | 100.0 | 48.6 |
| Abbreviations: ANC, antenatal care; CI, confidence interval; ITN, insecticide treated net | | | | | | | | | | | | | | | | | | | | | | | | |
